# Prevalence of Associated Anomalies in Cleft Lip and/or Palate Patients

**Published:** 2016-03

**Authors:** Shahin Abdollahi Fakhim, Nikzad Shahidi, Alireza Lotfi

**Affiliations:** 1 *Department of Otorhinolaryngology Head and Neck Surgery, Tabriz University of Medical Sciences, Tabriz, Iran.*

**Keywords:** Anomaly, Cardiac, Cleft, Oro-facial

## Abstract

**Introduction:**

Orofacial clefts are among the most common congenital anomalies. Patients presenting with orofacial clefts often require surgery or other complex procedures. A cleft lip or palate can be a single anomaly or a part of multiple congenital anomalies. The reported prevalence of cleft disease and associated anomalies varies widely across the literature, and is dependent on the diagnostic procedure used. In this study we determined the prevalence of associated anomalies in patients with a cleft lip and/or palate, with a specific focus on cardiac anomalies.

**Materials and Methods:**

In this cross-sectional study, 526 patients with a cleft lip and /or palate admitted to the children’s referral hospital between 2006 and 2011 were evaluated. All associated anomalies were detected and recorded. Patient information collected included age, gender, type and side of cleft, craniofacial anomalies and presence of other anomalies, including cardiac anomalies. Data were analyzed using SPSS version 16.

**Results:**

Of the 526 patients enrolled in the study, 58% (305) were male and 42% (221) were female. In total, 75% of patients (396) were aged between 4 and 8 years and 25% (130) were aged less than 4 years. The most common cleft type in our study was bilateral cleft palate. The most commonly associated anomaly among cleft patients, in 12% of cleft patients, was a cardiac anomaly. The most common cardiac anomaly was atrial septal defect (ASD).

**Conclusion:**

The prevalence of associated anomalies among orofacial cleft patients is high. The most common associated anomaly is cardiac anomaly, with ASD being the most common cardiac anomaly. There are no significant relationships between type of cleft and associated cardiac anomalies.

## Introduction

Orofacial clefts are among the most common congenital anomalies. Patients presenting with orofacial clefts often require various forms of surgery and other complex procedures. 

A cleft lip or palate can be a single anomaly or a part of multiple congenital anomalies (1). The reported prevalence of cleft disease and associated anomalies varies widely across the literature, and is dependent on the diagnostic procedure used (2). For example, in a study by Natsume et al., the prevalence of anomalies associated with cleft lip was 11.4% (3), versus 16.2% with a cleft lip and palate and 20.7% with a cleft palate only (4). Sarkozi et al. reported that 80% of cleft patients in their study had no associated anomalies, with only 20% reporting anomalies (5). In a study in 807 patients with a cleft lip or palate, Aljohar et al. reported that 238 cases had associated anomalies, 91 of which were cardiac anomalies (6). In a study in 460 neonates with cleft disease, Stoll et al. reported associated anomalies in 36.7% of patients. Associated anomalies were more common in cases of cleft palate only compared with other types of cleft. In their study, central nervous system (CNS) anomalies were the most common associated anomaly (7).

In another study in 1,293 neonates in South Korea with a cleft, Kim et al. reported that the prevalence of cleft lip was 34.1% and cleft lip and palate was 30.1% (8),while the prevalence of cleft palate was only 35.8%. Of these patients 5.4% had cardiac anomalies (9). Pavri et al. studied the demographics of orofacial clefts in Canada between 2002 and 2008. In their study, the prevalence of cleft lip was 17% compared with 41% for cleft palate and 42% for cleft lip and palate (10).

In a 2007 study by the Eurocat workgroup in the European Union, the overall incidence of cleft-associated anomalies was 29.2%. The incidence of associated anomalies among cleft lip patients was 36.6% and that of cleft lip and palate was 63.4%. In their study, musculoskeletal, cardiac and CNS anomalies were the most common anomalies (11). 

In another study in Sweden in 616 infants with cleft, Josef et al. found associated anomalies in 28% of patients with a cleft lip and palate and in 22% of patients with a cleft palate only, and in 18% of cleft lip cases. In their study, cardiac anomalies were the most commonly associated anomaly (12).

 Nancy et al. studied 282 cleft patients in England in 1987, and reported an overall incidence of cardiac anomalies among orofacial cleft patients of 6.7%. Associated cardiac anomalies included ventricular septal defect (VSD), and Tetralogy of Fallot (TOF) (13).

 Because of the apparent lack of information concerning the precise prevalence of various types of clefts and associated anomalies among cleft patients in Northwest Iran, we decided to undertake the current study. The goals of the study were to determine the prevalence of various types of cleft lip and palate, determine the incidence of associated anomalies among cleft patients, investigate if there is a significant relationship specifically between cleft diseases and cardiac anomalies (1-3).

## Materials and Methods

In this cross-sectional study, 526 patients with a cleft lip and/or palate admitted to the children’s referral hospital between 2006 and 2011 were evaluated. All patients were evaluated preoperatively by a neonatologist and pediatrician. All associated anomalies were detected and recorded.

Patient information collected included age, gender, type and side of cleft, craniofacial anomalies, and presence of anomalies including cardiac anomalies.Data were analyzed using SPSS version 16. A p-value less than 0.05 was considered significant.

## Results

**Fig 1:**
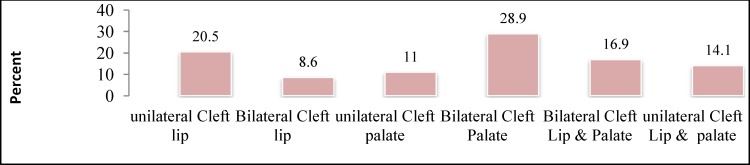
Types of cleft presented by the 526 patients enrolled in this study

Of the 526 patients enrolled in this study, 58% (n=305) were male and 42% (n=221) were female. In total, 75.3% of patients (n=396) were aged between 4 and 8 years and 24.7% (n=130) were aged less than 4 years. The most common cleft type was bilateral cleft palate (Fig.1).

Overall, 137 patients (28%) had an associated anomaly, of whom 84 were male and 53 were female. There was no significant relationship between gender and associated anomalies (P=0.386). 

**Fig 2:**
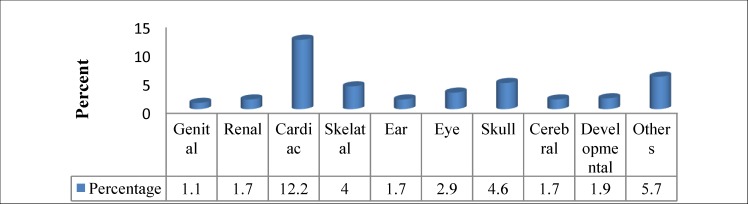
Distribution of anomalies among cleft patients

The most common associated anomaly among cleft patients was a cardiac anomaly, in 12.2% of cleft patients (Fig.2).

There was no significant relationship between cleft disease and associated anomalies (P=0.797). Among cleft patients with cardiac anomalies, 40 (7.6%) patients had atrial septal defect (ASD), 14(2.7%) had VSD, nine (1.7%) had mitral regurgitation (MR) and one (0.2%) had transposition of great arteries (TGA). Thus, the most common cardiac anomaly in our study was ASD. However, there was no significant relationship between cardiac anomalies and cleft disease (P=0.428). The distribution of type of cardiac anomaly according to type of cleft is presented in Table.1.

**Table 1:** Types of cardiac anomalies according to type of cleft

**Type**	**ASD**	**MR**	**TGA**	**VSD**
Unilateral cleft lip	14	1	0	2
Bilateral cleft lip	0	0	0	1
Unilateral cleft palate	2	2	0	1
Bilateral cleft palate	12	4	0	2
Bilateral cleft lip and palate	5	1	1	2
Unilateral cleft lip and palate	5	0	0	5
Others	2	1	0	1

There was no significant difference between type of cardiac anomaly and gender (Table.2).

**Table 2:** Type of cardiac anomalies according to gender

**Gender**	**ASD**	**MR**	**TGA**	**VSD**
Male	20	3	1	8
Female	20	6	0	6
				

## Discussion

In our study, the prevalence of associated anomalies among patients with orofacial clefts was 26%, which is less than that reported by Stoll et al. (7). 

 In our study, the most commonly associated anomaly was cardiac anomalies (12.2%), although the prevalence was lower than that reported by Nancy et al. in England (13), and Josef et al. in Sweden (12). 

In our study, most cardiac anomalies were in patients with bilateral cleft palate and in unilateral cleft lip. In the study by Sarkozi et al. in Hungry, the majority of cardiac anomalies were in patients with cleft palate only or cleft lip and palate. In our study, ASD was the most common cardiac anomaly (7.6%) (14). In another study in China, Sun et al. reported an overall prevalence of associated anomalies among orofacial cleft patients of 31.1%, with cardiac anomalies being the most common anomaly (45.1%) and ASD being the most common cardiac anomaly (15). 

In a Brazilian study in 220 patients, Barbosa et al. found a 9.5% prevalence of cardiac anomalies among cleft patients. The most common cardiac anomaly was mitral valve prolapse (MVP). Other anomalies in their study included ASD, PDA, VSD, TOF, and pulmonary valve stenosis(16). As in our study, the relationship between type of cleft and cardiac anomaly in this study was not significant.

## Conclusion

The prevalence of associated anomalies among orofacial cleft patients is high. The most common associated anomaly is a cardiac anomaly, with ASD being the most common cardiac anomaly. There are no significant relationships between type of cleft and associated cardiac anomalies. 
